# Difference Analysis of Ecological Vulnerability and Zoning Changes of National Energy and Chemical Bases Using FAHP Method

**DOI:** 10.3390/ijerph18136785

**Published:** 2021-06-24

**Authors:** Yue Zhang, Yue Chang, Kanhua Yu, Liyuan Zhang, Xuxiang Li

**Affiliations:** 1 School of Architecture, Chang’an University, Xi’an 710064, China; zhangyue-up@chd.edu.cn; 2 Shaanxi Provincial Academy of Environmental Science, Xi’an 710049, China; 3 School of Human Settlements and Civil Engineering, Xi’an Jiaotong University, Xi’an 712000, China; changyue@stu.xjtu.edu.cn (Y.C.); xxli@mail.xjtu.edu.cn (X.L.); 4 School of Water and Environment, Chang’an University, Xi’an 710064, China; zly2017@chd.edu.cn

**Keywords:** ecological vulnerability index (CEVI), geographic information system (GIS), fuzzy analytic hierarchy process (FAHP), NECB

## Abstract

Ecological vulnerability zoning research is an important basis for taking targeted regional ecological environment restoration and governance measures. This study analyzes the ecological vulnerability pattern and trend in the National Energy and Chemical Base (NECB) in the typical region of the Loess Plateau using GIS (Geographic Information System) data and the fuzzy analytic hierarchy process (FAHP) approach. Based on the human activity–natural environment factor index system, 13 factors representing human activities, socioeconomics, meteorology, soil and topography are selected to build an ecological vulnerability index (EVI) system in the NECB region, which aims at identifying the regional features of eco-environment and major environmental problems in the Loess Plateau. By calculating ecological vulnerability zoning, a model of ecological vulnerability trend change is constructed to quantitatively study the overall temporal and spatial variation of ecological vulnerability. The results indicate that the medium and heavy levels of ecological vulnerability index were mostly distributed in the areas with developed energy and chemical industries, and the slight and light levels were distributed in the southern area and developed agricultural regions. A comprehensive ecological vulnerability index had a score of 2.3207 in 2015 and 2.441 in 2000, indicating that the ecological security gradually improved. Nevertheless, highly intense human activities accelerated the degradation of regional eco-environment in recent years.

## 1. Introduction

Ecological vulnerability is a specific property of an ecosystem, which refers to the ability of an ecosystem to recover from external disturbances in a certain area. National Energy and Chemical Bases are areas in China that are based on and characterized by the development of energy and related chemical industries. Under the influence of external forces, mainly wind and running water, the desertification and soil erosion in the NECBs are intensifying, and the sand dunes in the north are stretched, forming a wind–sand landform, mainly dominated by wind erosion. The southern terrain is fragmented, and the gullies are vertical and horizontal, forming hilly and gully loess landforms dominated by water erosion. A large amount of silt discharge flows through the Yellow River here, which is a typical loess landform area. It has become one of the most serious soil erosion areas, and the ecological environment is extremely vulnerable. Then, excessive exploitation for mineral resources has deteriorated the fragile ecological conditions in this area. Taking effective measures to control the deterioration of its ecological environment can protect China’s energy and chemical base and the safety of the Yellow River downstream. It is hence necessary to evaluate ecological vulnerability of the NECB region for ecological environmental protection and governance.

Most of the research on ecological vulnerability is status research, but the research on ecological vulnerability zoning is insufficient. A variety of evaluation methods have been developed, such as Principal Component Analysis [1,2,3], the fuzzy evaluation method [4,5], and the entropy method. However, as the multi-index evaluation system involves a wider range of content, most of the required data have spatial data and part of the data are historical data, which are difficult to obtain [6,7]. The analytic hierarchy process (AHP) is a reasonable and feasible evaluation method that combines qualitative and quantitative information by decomposing major problems into different systematic hierarchies [8]. However, the AHP method makes it very difficult to check whether the matrix is consistent. The entropy method ignores the importance of the indicators themselves, and sometimes the determined indicator weights can be far from the expected results. The fuzzy analytic hierarchy process (FAHP) can deal with this shortcoming better, so this paper uses the FAHP method for ecological vulnerability evaluation of the NECB area.

In order to obtain a reasonable ecological vulnerability assessment, a geographic information system (GIS) was used with the FAHP model [9,10]. By using GIS, multiple layers of information can be integrated in different combinations. This provides an effective tool that can analyze numerous spatial parameters involved in ecological vulnerability [11,12,13,14]. The evaluation system includes natural factors, environmental factors and socio-economic factors. Based on this ecological vulnerability assessment model, ecological vulnerability zoning and the comprehensive ecological vulnerability index (CEVI) were computed for the study area. The purpose was to quantitatively study the changing status of ecological vulnerability in the region and clarify the changing trend of regional ecological vulnerability. The assessment results were mapped to show spatial and temporal distributions of ecological vulnerability zoning in the whole study area, which provided a feasible way of thinking for the regional study of regional ecological environment instability.

## 2. Study Area and Data Acquisition

### 2.1. Description of Study Area

**Figure 1:**
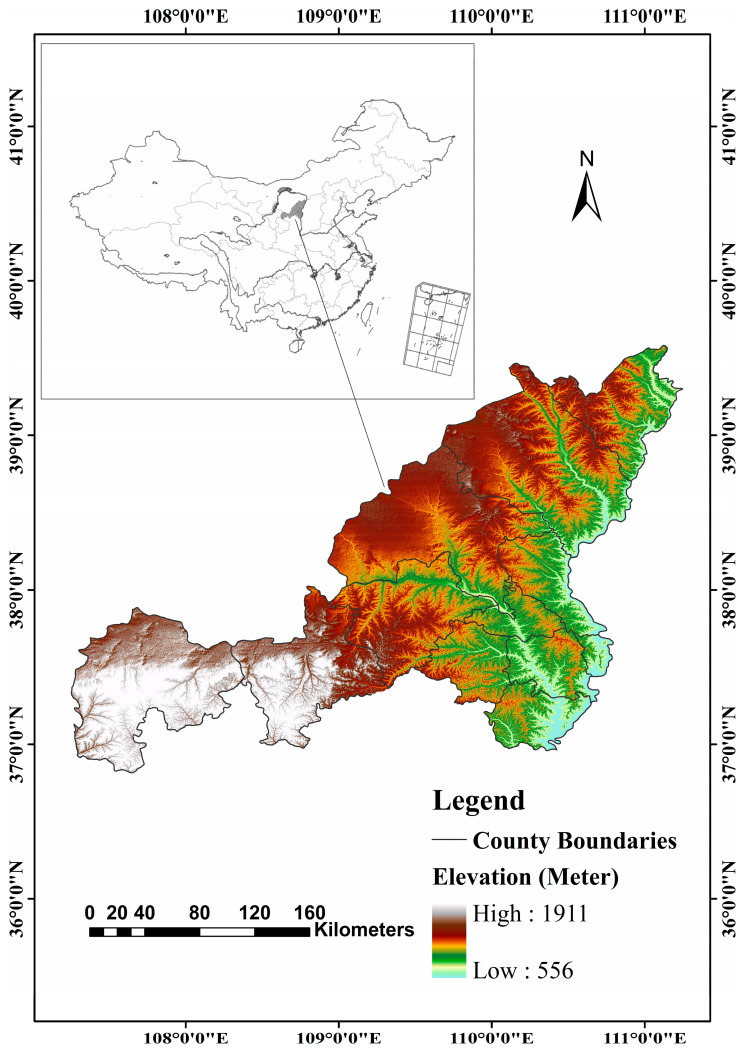
Location and elevation of the study area.

The NECB is located at 36°57′ to 39°34′ N and 108°28′ to 111°15′ E. It is located in the northernmost part of Shaanxi Province, and belongs to the transition region from Maowusu sandy land to the Loess Plateau region of northern Shaanxi in terms of geomorphologic types (See Figure 1).

It belongs to the semi-arid grassland climate in the continental monsoon region of the mid-temperate zone, with an average annual precipitation of 316.4 mm and uneven spatial and temporal distribution. More than 70% of the annual precipitation is concentrated from June to September, with heavy rain, large annual variability and fragile habitat characteristics.

The study area is rich in mineral resources, and is a highly rich area of coal, natural gas, oil, salt and rock resources in China. The unique natural conditions and resource reserves make it not only one of the cities with the most serious soil erosion in China, but also one of the cities with the most drastic land use/cover changes in the past 20 years. The NECB now has jurisdiction over 11 counties and 1 district, with a total area of about 43,578 km^2^. It is very necessary to study the ecological vulnerability zoning in this region, which is of great significance to realize the regional sustainable development.

### 2.2. Data Acquisition

Regional ecological vulnerability assessment is a typical multi-indicator comprehensive system, which can be characterized by climate, topography, vegetation, social and economic factors. These factors are mainly derived from satellite remote sensing images, the statistical yearbook and its bulletin. The evaluation indexes all have spatial geographic attributes, so surface data can be obtained by point data interpolation. Remote sensing data and DEM (Digital Elevation Model) came from the Geospatial Data Cloud (http://www.gscloud.cn/ accessed on 30 March 2021).

1: Remote sensing images (Landsat 4-5TM and Landsat 8 OLI_TIRS) were mainly used to interpret vegetation coverage and land use degree;
2: DEM data could be used to extract slope and elevation data. The above data were all non-point source raster data, while the socio-economic data and meteorological data were all point source data;
3: Agricultural output, population density, and GDP (Gross Domestic Product) per capita were acquired from Statistical Yearbook of Shaanxi Province and Yulin in 2000, 2008 and 2015;
4: Precipitation, annual average temperature and soil erosion data were provided by Shaanxi Meteorological Bureau.

## 3. Methodology

### 3.1. Evaluation Principles and Influencing Factors

#### 3.1.1. Evaluation Principles

The leading factors of ecological vulnerability in specific regions are different, so the index system constructed should reflect its characteristics. Due to the limitation of data acquisition methods and accuracy, there is no unified evaluation system for ecological vulnerability indicators [2,3]. Constructing an evaluation system that can reflect the characteristics of ecological vulnerability in typical regions and making it representative is the key content of evaluation research. The local characteristics of the ecological environment are the basis of ecological vulnerability assessment, based on the quantitative screening of local data of the ecological environment [15]; we considered all possible environmental variables for the present assessment.

#### 3.1.2. Influencing Factors

Natural factors include elevation, rainfall, temperature, slope and hours of sunshine [16,17]. Environmental factors include land use types, vegetation coverage and soil erosion [18,19]. The evolution of ecological vulnerability in characteristic regions is largely influenced by the factors of human activities, which are closely related to the factors of social and economic activities [20,21]. Natural population growth rate, agricultural output, population density, reforestation area and per capita GDP were, therefore, selected to evaluate the impacts of human activities.

### 3.2. Standardization of Factor Index

In a multi-index evaluation system, due to the different nature of different indexes, they have different dimensions and orders of magnitude. When the level of each index differs greatly, it will highlight the role of the index with a higher value in the comprehensive analysis and relatively weaken the role of the index with a lower value. Therefore, in order to ensure the reliability of the results, it is necessary to standardize the original index data. The original values were standardized in the following formula.

(1) $$X=\frac{X_{i}-X_{\min}}{X_{\max}-X_{\min}}$$

where *i* is the evaluation unit, *X_i_* is the original value of *i*, *X*_max_ and *X*_min_ are the maximum and minimum values of *i*.

### 3.3. Weight of Evaluation Factors

The FAHP method converts these evaluations into manageable values. Since it decomposes the complex problem into some levels and factors, it can well reflect the slight importance and obvious importance, and the complex multi-indicator system can be quantitatively analyzed and processed by the FAHP method, only that the subjective factors are large and difficult to be controlled by human. As it can assign proper weights to various factors, an ecological evaluation system is suitable for the use of the FAHP method [22,23]. Based on the Delphi expert advice system, this study used the FAHP method to determine the weight of each factor [24]. The main analytic process was as follows (see Table 1).

Based on expert advice, the assessment model was divided into three levels—A, B and C (see Table 2). If factor *a* is preferred to factor *b* and factor *b* to factor *c*, then factor *a* must be more preferred to factor *c*. Generally, the consistency ratio (*CR*) is used to indicate the probability that the matrix judgments were randomly generated [26,27]:

(2) $$CR=\frac{CI}{RI}$$

where *RI* is the average of the resulting consistency index depending on the order of the matrix given by Saaty [27], and consistency index (*CI*) is defined as:

(3) $$CI=\frac{\lambda_{\max}-n}{n-1}$$

where *λ*_max_ is the largest or principal eigenvalue of the matrix, and *n* is the order of the matrix. When *CR* was less than 0.10, the matrix had a reasonable consistency [27]. In this study, the *CR* is 0.03, and it is acceptable. Finally, the weights for all the factors were determined, as shown in Table 2. A is the ecological vulnerability index, and B1, B2 and B3 are the natural factors, environmental factors and socio-economic factors, respectively.

### 3.4. Environmental Vulnerability Index (EVI) Calculation

In this multi-index evaluation system, values of all factors were overlaid, and the comprehensive evaluation value was used to reflect the degree of the ecological vulnerability. Therefore, the comprehensive evaluation value was the sum of the corresponding weight values of all selected factors by using the following formula:

(4) $$EVI=\sum_{i=1}^{13}x_{i}y_{i}$$

where *EVI* is the ecological vulnerability index, *x_i_* is the weight of factor *i* and *y_i_* is the normalized value of factor *i*. The higher an *EVI* value is, the more vulnerable the ecological environment is.

### 3.5. Determination of Ecological Vulnerability Zoning Standards

**Figure 2:**
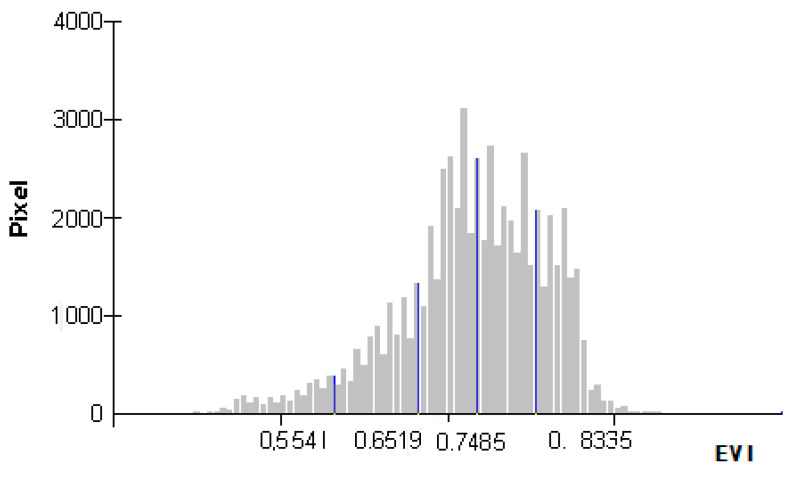
Data distribution histogram of the ecological vulnerability index in NECB.

In the usual study of ecological vulnerability, the ecological vulnerability index is the only index used to measure the state of evaluation units. However, the EVI data distribution interval is wide, and its size change cannot directly reflect the change of regional ecological vulnerability. For regional ecological environmental management, EVI zoning can present the characteristics of regional ecological vulnerability more objectively, because dividing the study area can be more specific to understand the detailed changes of the area and can observe the characteristics of the area more directly and objectively. In this study, natural break classification (NBC) was used to rationally classify the ecological vulnerability of the whole region using ArcGIS 9.3 software [28]. This method was used to classify the computed results by analyzing the histogram of ecological vulnerability index distribution (Figure 2), and the results of ecological vulnerability assessment can be divided into five grades—potential, slight, light, medium and heavy vulnerability levels (Table 3).

### 3.6. Analysis of the Vulnerability Trends

In order to make a quantitative analysis of the trend on ecological vulnerability from 2000 to 2015, a comprehensive index representing vulnerable situations was built. Based on the vulnerability ranks, every grade is granted a quantified value, respectively (Table 3). The formula for defining the comprehensive ecological vulnerability index (CEVI) is shown as below.

(5) $$CEVI_{j}=\sum_{i=1}^{n}S_{i}\times \frac{N_{i}{}_{{}_{}}}{M_{j}}$$

In this formula, *n* is the number of valuation grade, *CEVI_j_* is the comprehensive ecological vulnerability index of unit *J*, *N_i_* is the occupied area of grade *i* in analysis unit *j*, *M_j_* is the area of analysis unit *j*, and *S_i_* is the graded value of grade *i*. In general, the whole trend can be worked out by comparing the ecological vulnerability index (CEVI) values of each period and the distribution of each level.

## 4. Results

### 4.1. Changes in Natural, Ecological and Socio-Economical Factor Indexes

**Figure 3:**
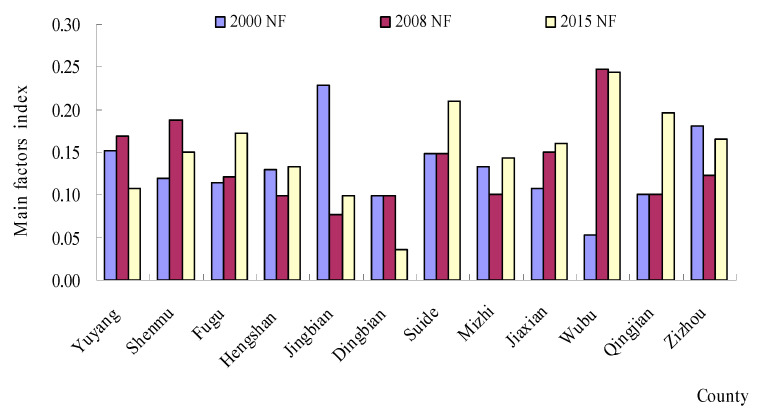
The changes in natural factors (NFs) from 2000 to 2015.

**Figure 4:**
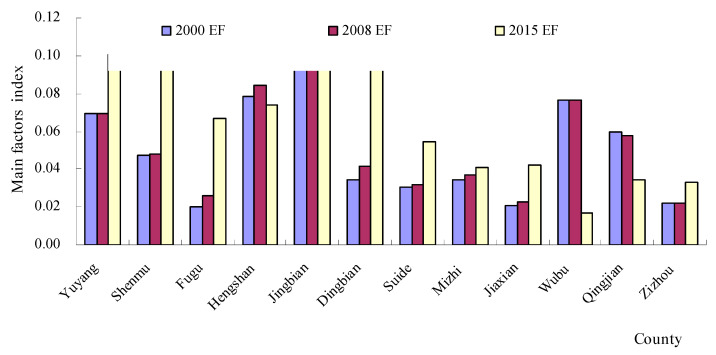
The changes in environmental factors (EFs) from 2000 to 2015.

**Figure 5:**
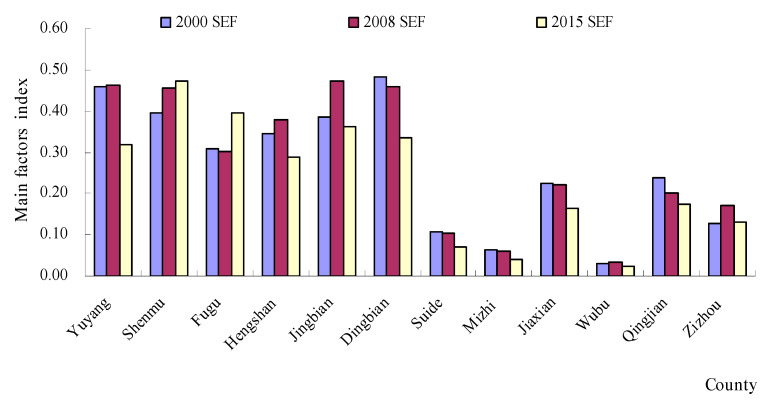
The changes in socio-economic factors (SEFs) from 2000 to 2015.

The area statistics for natural factors (NFs), environmental factors (EFs) and socio-economic factors (SEFs) are shown in Figure 3, Figure 4 and Figure 5. The evaluation results showed that the NFs and SEFs were the major factors affecting the ecological vulnerability index in most of the counties from 2000 to 2015. In six northern counties, the SEF index was much larger than the EF index and NF index in 2000. In addition, a similar law also occurred in 2008 and 2015. However, the NF index was much larger than the EF index and SEF index from 2000 to 2015. From Figure 3, we can see that after 2008, the highest NF region is Wubu, and the lowest is Dingbian, while the EF index (Figure 4) is higher in 2000, 2008 and 2015, and lower in Jingbian and Zizhou. The region in Figure 5 with the highest SEF index for essentially all three years is Shenandoah, and the region with the lowest is Wubu.

The spatial distribution of the three major factors (Figure 3, Figure 4 and Figure 5) reveals significant geographical variations. The SEF index values in the northern regions were larger than those in the southern regions. However, the NF index in the southern regions was the main factor affecting the ecological vulnerability, where there was plenty of rain. These regions were the prime agricultural bands with high biotransformation ratios, which have the majority of the cultivated land in the NECB.

### 4.2. Study on Zoning of Ecological Vulnerability

**Figure 6:**
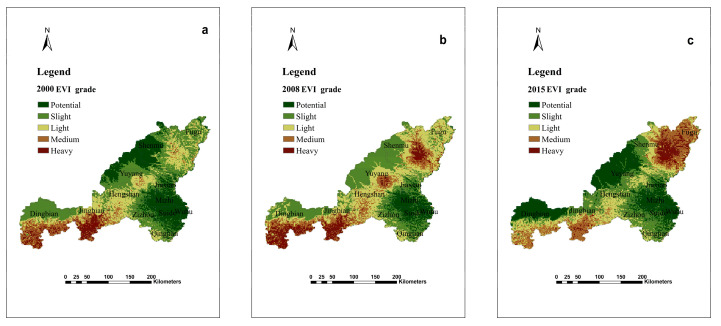
The distribution of ecological vulnerability index in NECB in 2000 (**a**), 2008 (**b**) and 2015 (**c**).

According to the ecological vulnerability index value, the ecological vulnerability of the NECB area was partitioned by using the natural break classification, and it was divided into five categories according to the corresponding standards, namely potential vulnerability, slight vulnerability, light vulnerability, medium vulnerability and heavy vulnerability. In addition, ArcGIS 9.3 software was used to calculate the area of the ecological vulnerability partition of NECB area, and the partition results could better reflect the local actual ecological conditions (Figure 6 and Table 4).

According to the results of ecological vulnerability zoning, ecological vulnerability could be clearly reflected. In 2000, slight vulnerability zones accounted for the largest area, accounting for 30.89% of the total area, followed by potential vulnerability areas, accounting for 26.68% of the total area. Meanwhile, the area of heavy vulnerability was the smallest, accounting for about 5.88% of the total area. In 2008, the area of slight vulnerability remained the largest with a percentage of 35.86%, followed by 30.25% of light vulnerability. Similarly, the area of heavy ecological vulnerability was still the smallest, but the proportion had increased to 9.52%. In 2015, the zoning pattern of ecological vulnerability was similar to that in 2000. The area occupied by slight vulnerability areas was still the largest, about 37.95%, and the area occupied by heavy vulnerability areas was still the smallest, and the area decreased to about 5.50% compared with that in 2005.

### 4.3. Changes of the Ecological Vulnerability Index with Administrative Regions

From 2000~2015, the ecological vulnerability index in the most of administrative regions decreased in the NECB, indicating that the ecological environment gradually stabilized. In some counties, however, ecological vulnerability index increased gradually. The ecological vulnerability index of Yuyang District, Shenmu County, Fugu County and Jingbian County were abnormal, where the EVI of the latter stage was larger than that in the earlier stage. The EVI in the six southern counties gradually became smaller from 2000 to 2015, suggesting that the security of ecological environment increased gradually.

Among them, the EVI of the six northern counties (including Yuyang District, Fugu County, Shenmu County, Hengshan County, Jingbian County and Dingbian County) was significantly higher than the six southern counties (including Suide County, Mizhi County, Jiaxia County, Qingjian County, Wubu County and Zizhou County). In other words, the environment safety gradually become stronger from north to south, and then such incremental changes were closely related to rainfall and socio-economic development.

The ecological vulnerability indexes of Mizhi County and Wubu County were the lowest, which can illustrate that regional ecological security was highest in this region. In all twelve counties, the ecological vulnerability indexes of Jingbian County and Yuyang District were higher than other counties and urbanization expansion was one of the important factors.

**Figure 7:**
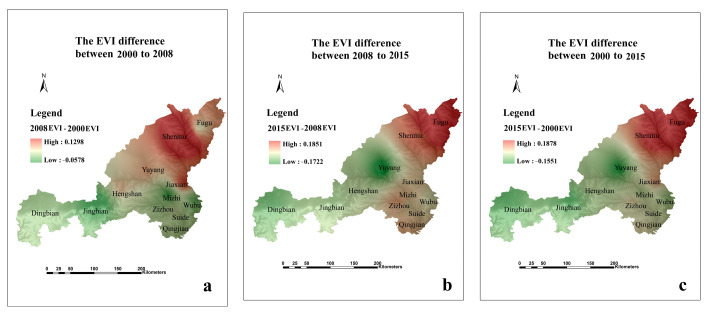
The EVI changes between 2000 and 2008 (**a**), between 2008 and 2015 (**b**), between 2000 and 2015 (**c**).

From 2000 to 2008, the EVI in most regions gradually decreased, which also reflected that ecological environment gradually stabilized. However, the EVI in northern counties (including Fugu County, Shenmu County and Yuyang District) increased (see Figure 7). Overall, the EVI in most regions gradually decreased, but the EVI in Fugu County, Shenmu County and Jiaxian County gradually increased from 2000 to 2015.

### 4.4. Comprehensive Ecological Vulnerability Changes in NECB

The change of the comprehensive ecological vulnerability index (CEVI), shown in Table 4, is analyzed. The situation in 2015, with the CEVI being 2.3207, was better than in 2000, with the CEVI being 2.441, and the latter was better than in 2008, with the CEVI being 2.7722. The bigger the value of CEVI is, the more serious ecological vulnerability is. During this period, the ecological vulnerability was also closely correlated with energy development in the National Energy and Chemical Base. The medium and heavy levels of EVI were mostly distributed in the areas with developed energy and chemical industries. The heavy vulnerability level was entirely distributed in the area with an energy mining industry, whereas the slight and light level was distributed in the southern area and agriculture developed regions. This finding indicates that the ecological vulnerability increases with energy economic development, which could reflect the harsh environmental conditions at the energy mining industry.

## 5. Discussion

### 5.1. Analysis of Ecological Vulnerability Assessment

Located at the junction of the Loess Plateau and the Maowusu Desert, the NECB is a typical area of soil and water loss in China and a key area of mineral energy development in China, with poor ecosystem stability. According to the analysis chart, the NECB’s medium and heavy ecological vulnerability areas accounted for more than 20% of the total area from 2000 to 2015. The percentage of intermediate buffer zone increased from 16.90% in 2000 to 22.39% in 2015. Its variability was relatively prominent, which could better reflect the ecological environment of the study area.

In 2000, the northern part of the study area was characterized by potential and mild ecological vulnerability, while the southern area’s ecological vulnerability was classified as moderate and severe. In 2015, moderate and severe EVI appeared in the northern part of the study area, while the southern part was classified as having mild ecological vulnerability (Figure 6 and Figure 7). Energy extraction and transitional grazing have caused the region’s ecological vulnerability to deteriorate from 2000 to 2008. The local government implemented a policy of returning farmland to forests in 1999, and the ecological vulnerability has improved.

This result showed that the socio-economic factors were the main force leading to the change in the regional ecological vulnerability. However, Wang et al. [18] analyzed the ecological vulnerability in the Tibetan Plateau and thought that the ecological vulnerability was also closely correlated with altitude. As the average elevation of the Tibetan Plateau is 4000~5000 m, alpine hypoxia directly reduces the effect of human activities on the ecological environment. Thus, the natural environment, especially altitude, was the main factor affecting the ecological vulnerability in the Tibetan Plateau. The study region was the National Energy and Chemical Base. Therefore, a larger number of human activities affected the ecological social and economic vulnerability. These findings were confirmed by our field investigation and consistent with the actual environmental situation, which demonstrated that the evaluation results represented regional features and status.

### 5.2. Effects of Main Driving Forces on Ecological Vulnerability

**Figure 8:**
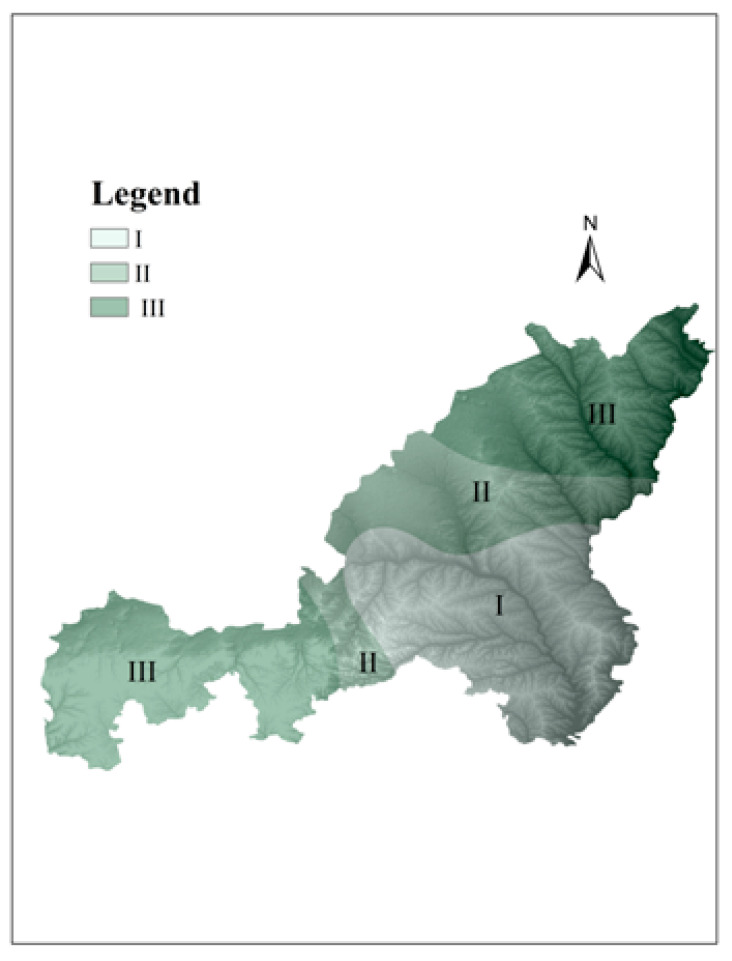
The zone of the different driving factors.

In the NECB area, the ecological vulnerability index in most regions decreased gradually, which reflected that the ecological quality had been improved. However, ecological vulnerability became worse in some counties in the past ten years. From 2000 to 2015, the economic factor, urbanization development factor, policy factor and natural environmental factors were the driving forces affecting ecological vulnerability changes. Natural environmental factors were the common constraints which, along with the extensive development of human society, exacerbated ecological vulnerability in the NECB area of the Loess Plateau. Different socio-economic driving factors affected the specific zones (see Figure 8). Energy exploitation mainly affected zone III. The urbanization factor was mainly reflected in zone II. Zone I was mainly distributed in the developed agriculture region, and the policy of returning farmland to forest also had an effect on this region.

Energy exploitation was an important driving force affecting the ecological environmental balance, especially in ecologically fragile areas. A wide range of energy exploitation not only destroyed the surface vegetation, but also caused surface subsidence and soil erosion. The energy exploitation of regions, including Dingbian County, Shenmu County, and Fugu County, was mainly distributed in zone III. The ecological vulnerability level of this zone was heavy. This area was the National Energy and Chemical Base, and economic development mainly depends on energy and chemical industries. Extensive energy exploitation patterns would inevitably lead to the expansion of ecological vulnerability regions. Necessary control measures should be taken, otherwise it would affect sustainable development in the National Energy and Chemical Base.

Urbanization development mainly affected zone II, including Yuyang District, Hengshan County and Jingbian County. Due to the relative concentration of population, urban expansion required that a lot of land was converted into building land and industrial land. Most of these regions were at medium level, but ecological vulnerability index was relatively low, and the vulnerability was light or slight in the suburban area. From 2000 to 2015, this change was more obvious, and the main reason may be related to the construction of shelter belts around the city because of the implementation of the policy of returning farmland to forest.

According to the social, economic and natural environment of the NECB, agriculture is the leading industry in zone I, lacking the support of the energy and mineral development industries. The main sub regions include Mizhi County, Suide County, Jia County, Qingjian County, Wubu County and Jia County. The ecological vulnerability of zone I was mainly composed of potential ecological vulnerability and slight ecological vulnerability, and the ecological environment was in good condition. In addition, due to the aggravation of soil erosion in the Loess Plateau, the project of returning farmland to forest and grass was implemented in 1999, which promoted the gradual restoration of ecological environment. In the past decade, the sloping land that was not suitable for cultivation had been gradually transformed into grassland and forest land [29]. In general, the comprehensive ecological vulnerability index decreased from 2.4441 in 2000 to 2.3207 in 2015 (Table 4), reflecting the gradual improvement of ecological environment stability.

## 6. Conclusions

This study proposed a method considering multiple factors for assessing the regional ecological vulnerability, using GIS and the FAHP to closely reflect the real situation of the NECB area. The ecological vulnerability index and its change were evaluated and analyzed for the typical hilly–gully region in the Loess Plateau.

In 2000~2015, the ecological vulnerability in the NECB was at the slight level, and areas with potential, slight and light vulnerability approximated to 4/5 of the total area (Table 4). Moreover, the ecological vulnerability decreased from 2000 to 2015 as the CEVI value decreased from 2.4410 in 2000 to 2.3207 in 2000. Based on the results, they can reflect that the ecological quality has gradually improved in most places in the past 15 years.

Overall, the ecological quality has gradually improved in most places in the past 15 years. The ecological vulnerability index of Yuyang District, Shenmu County, Fugu County and Jingbian County were abnormal, where the EVI increased over the years. The extremely vulnerable level was entirely distributed in the energy industry, whereas the slight and light levels were distributed in the southern area and agriculture developed regions.

Through this method, the ecological vulnerability of special regions can be better divided, and the trend of ecological vulnerability can be better grasped, which can provide reference for other regions to adopt targeted ecological and environmental policies.

## Figures and Tables

**Table 1:** Scale of binary comparison [25].

Degree of Importance	Definition
0.5	Equal importance of two elements.
0.6	Weak importance of an element in comparison to the other one.
0.7	Strong importance of an element in comparison to the other one.
0.8	Certified importance of an element in comparison to the other one.
0.9	Absolute importance of an element in comparison to the other one.
0.1, 0.2, 0.3, 0.4	If element *i* and element *j* are compared with each other to obtain *a*, the judgment of element *j* and *i* element is *b*.

**Table 2:** Weights of factors for environmental vulnerability evaluation in NECB.

First Level	Second Level	Weight	Third Level	Weight
A	B1	0.3196	C1	0.0216
			C2	0.1443
			C3	0.0398
			C4	0.1007
			C5	0.0133
	B2	0.1220	C6	0.0291
			C7	0.0762
			C8	0.0166
	B3	0.5584	C9	0.0460
			C10	0.1457
			C11	0.2618
			C12	0.0728
			C13	0.0321

Note: C1—elevation, C2—precipitation, C3—annual average temperature, C4—25° above the slope, C5—annual sunshine time, C6—farmland area, C7—forest cover rate, C8—soil and water loss area, C9—natural population growth rate, C10—agricultural/industrial output ratio, C11—population density, C12—afforestation, C13—GDP per capita.

**Table 3:** Ecological vulnerability index (EVI) classification in NECB.

Grade Level	Evaluation Level	Ecological Vulnerability Index
1	Potential vulnerability	<0.5541
2	Slight vulnerability	0.5541~0.6519
3	Light vulnerability	0.6519~0.7485
4	Medium vulnerability	0.7485~0.8335
5	Heavy vulnerability	>0.8335

**Table 4:** The proportion of each EVI level and the result of comprehensive ecological vulnerability index (CEVI).

Year	EVI Grade	Area Percent	Comprehensive Ccological Vulnerability Index (CEVI)
2000	Potential	26.68%	2.4441
	Slight	30.89%	
	Light	19.64%	
	Medium	16.90%	
	Heavy	5.88%	
2008	Potential	10.00%	2.7727
	Slight	35.86%	
	Light	30.25%	
	Medium	14.62%	
	Heavy	9.26%	
2015	Potential	25.05%	2.3207
	Slight	37.95%	
	Light	22.39%	
	Medium	9.11%	
	Heavy	5.50%	

## Data Availability

No new data were created or analyzed in this study. Data sharing is not applicable to this article.
